# The effects of dietary supplementation with inulin and inulin‐propionate ester on hepatic steatosis in adults with non‐alcoholic fatty liver disease

**DOI:** 10.1111/dom.13500

**Published:** 2018-09-16

**Authors:** Edward S. Chambers, Claire S. Byrne, Annette Rugyendo, Douglas J. Morrison, Tom Preston, Catriona Tedford, Jimmy D. Bell, Louise Thomas, Arne N. Akbar, Natalie E. Riddell, Rohini Sharma, Mark R. Thursz, Pinelopi Manousou, Gary Frost

**Affiliations:** ^1^ Section for Nutrition Research, Faculty of Medicine Imperial College London, Hammersmith Hospital London UK; ^2^ Stable Isotope Biochemistry Laboratory Scottish Universities Environmental Research Centre, University of Glasgow Glasgow UK; ^3^ School of Science University of the West of Scotland Hamilton UK; ^4^ Department of Life Sciences, Faculty of Science and Technology, Research Centre for Optimal Health University of Westminster London UK; ^5^ Division of Infection and Immunity University College London London UK; ^6^ Faculty of Health and Medical Sciences University of Surrey Guildford UK; ^7^ Department of Surgery and Cancer Imperial College London London UK; ^8^ Liver Unit St Mary's Hospital, Imperial College NHS Trust London UK

**Keywords:** clinical trial, dietary intervention, fatty liver, insulin resistance

## Abstract

The short chain fatty acid (SCFA) propionate, produced through fermentation of dietary fibre by the gut microbiota, has been shown to alter hepatic metabolic processes that reduce lipid storage. We aimed to investigate the impact of raising colonic propionate production on hepatic steatosis in adults with non‐alcoholic fatty liver disease (NAFLD). Eighteen adults were randomized to receive 20 g/d of an inulin‐propionate ester (IPE), designed to deliver propionate to the colon, or an inulin control for 42 days in a parallel design. The change in intrahepatocellular lipid (IHCL) following the supplementation period was not different between the groups (*P* = 0.082), however, IHCL significantly increased within the inulin‐control group (20.9% ± 2.9% to 26.8% ± 3.9%; *P* = 0.012; *n* = 9), which was not observed within the IPE group (22.6% ± 6.9% to 23.5% ± 6.8%; *P* = 0.635; *n* = 9). The predominant SCFA from colonic fermentation of inulin is acetate, which, in a background of NAFLD and a hepatic metabolic profile that promotes fat accretion, may provide surplus lipogenic substrate to the liver. The increased colonic delivery of propionate from IPE appears to attenuate this acetate‐mediated increase in IHCL.

## 1 INTRODUCTION

Non‐alcoholic fatty liver disease (NAFLD), a condition characterized by the accumulation of fat within the liver, is regarded as a major risk factor in the development of type 2 diabetes.^1^ The prevalence of NAFLD is strongly associated with obesity,^1^ thus current guidelines for the prevention and management of NAFLD are based solely on body weight loss through diet and exercise.^1^ While lifestyle modifications are successful in reducing body weight in the short term, numerous studies show that long‐term maintenance of body weight loss in obese individuals is very poor.^2^ Lifestyle modifications alone are therefore unlikely to reduce the growing prevalence of NAFLD and there is an urgent need to develop therapeutic interventions that can safely be applied at the population level.

Recent investigations suggest that diet, the gut microbiota and liver fat storage could be linked through a mechanism involving short chain fatty acids (SCFA), the major products of dietary fibre fermentation in the colon. It has been repeatedly observed that when animals are fed fermentable fibre they are protected against steatosis induced by high fat diets.^3^, ^4^, ^5^ This effect may be because of the SCFA propionate, as ~90% of propionate produced in the colon is extracted from the portal vein by the liver,^6^ which has been shown to alter hepatic metabolic processes to reduce lipid content.^7^, ^8^ To augment colonic propionate production we have developed an inulin‐propionate ester (IPE), whereby the SCFA propionate is bound to the dietary fibre inulin, which is released through microbial hydrolysis in the colon.^9^ Our recent first‐in‐human studies provided preliminary evidence that supplementing the diet with 10 g/d IPE for 24 weeks reduced liver fat content in adults with NAFLD.^9^ These volunteers were identified as having NAFLD on the basis of an elevated intrahepatocellular lipid (IHCL) content from magnetic resonance imaging.

The aim of the current study was to develop in vivo proof‐of‐concept for IPE as a therapeutic to reduce hepatic steatosis in volunteers with a histological confirmation of NAFLD, which is considered the gold standard to establish diagnosis. We hypothesized that the addition of 20 g IPE to the diet of adults with NAFLD for 42 days would significantly reduce IHCL compared with 20 g of an inulin control.

## 2 METHODS

All volunteers provided informed, written consent prior to the clinical trial which was approved by the London Brent Research Ethics Committee (14/LO/0645). The study was carried out in accordance with the Declaration of Helsinki and is registered with the ISRCTN registry (ISRCTN71814178). A detailed methodology is presented in File S1 for this article. Men and women aged 18 to 65 years, with a body mass index (BMI) of 20 to 40 kg/m^2^ were recruited from liver clinics at St Mary's Hospital, Imperial College Healthcare National Health Service Trust. Potential volunteers were eligible if they had a confirmation of NAFLD by liver biopsy within the previous 5 years and controlled blood glucose levels (HbA1c < 48 mmol/mol). The study was conducted using a randomized, double‐blind, placebo‐controlled, parallel design. Subjects received either 20 g/d of inulin control or IPE for 42 days. The 20 g dose of IPE would have provided 14.6 g of inulin (and 5.4 g bound propionate) to the diet.^9^ Inulin was therefore chosen as a positive control to account for any effects that may derive from fermentation of this substrate by the gut microbiota. The supplements were provided to volunteers in 10 g ready‐to‐use sachets and they were instructed to mix the contents into their habitual diet twice a day. Participants were required to attend the NIHR Imperial Clinical Research Facility pre‐ (day 0) and postsupplementation (day 42) to determine outcome measures.

## 3 RESULTS AND DISCUSSION

**Figure 1:**
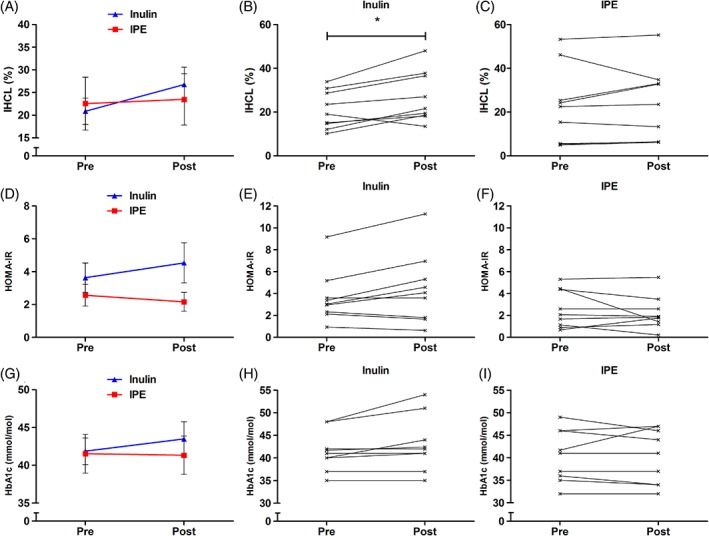
Effects of 42 days of inulin control and inulin‐propionate ester (IPE) supplementation on liver fat and glucose homeostasis. A to C, Intrahepatocellular lipid (IHCL); D to F, homeostatic model assessment of insulin resistance (HOMA‐IR); and G to I, glycosylated haemoglobin (HbA1c). Group data (A, D and G) are expressed as mean ± SEM (n = 9)

Of 20 volunteers that were randomized and enrolled into the study, data were analysed from the 18 volunteers that completed the supplementation period (Figure S1, File S1). The characteristics of these volunteers are presented in Tables 1 and S1 (File S1). Estimated compliance was similar in the supplementation groups (inulin control: 90% ± 7% vs. IPE: 95% ± 2%; *P* = 0.213). The changes in IHCL (Figure 1A‐C and Table 1) were unexpected, as we observed an increase in IHCL postsupplementation in both groups (main effect for time; *P* = 0.020). The change in IHCL was not significantly different between supplementation groups (Figure 1A; *P* = 0.082), however, within‐group analysis showed that IHCL was significantly increased within the inulin‐control group (Figure 1B; *P* = 0.012) and not the IPE group (Figure 1C; *P* = 0.635). Analysis of metabolic and inflammatory responses (Figure 1D‐I, Figure S2; Tables S2‐S4, File S1) highlights that the change in insulin resistance (HOMA‐IR) was significantly different between groups (Figure 1D; *P* = 0.046), with a non‐significant increase in the inulin‐control group (Figure 1E; *P* = 0.060) and decrease in the IPE group (Figure 1E; *P* = 0.389), respectively. There were no within‐ or between‐group differences in body composition (Table 1), self‐reported food intake or physical activity following the supplementation period (Table S5, File S1). Our hypothesis was that IPE supplementation would decrease IHCL in adults with NAFLD, as observed in our previous study^9^; however, IPE supplementation did not reduce liver fat content. The disparate outcome may be explained by methodological differences in IPE dose (10 g/d vs. 20 g/d) and exposure (6 weeks vs. 24 weeks) in the two studies. Furthermore, volunteers in the current study had a confirmation of NAFLD by liver biopsy, which is considered the gold standard to establish diagnosis, and metabolic parameters would indicate these individuals had poorer glycaemic control compared with the volunteers from our previous study (fasting glucose: 5.0 mmol/L vs. 6.1 mmol/L; HbA1c: 38 mmol/mol vs. 42 mmol/mol).

**Table 1:** Baseline characteristics of volunteers and changes in intrahepatocellular lipid and body composition following 42 days of inulin control or inulin‐propionate ester (IPE) supplementation

	Inulin control			IPE			Mixed ANOVA	
	(n = 9)			(n = 9)			Time	Group × Time
Variable	Pre	Post	*P* value	Pre	Post	*P* value	*P* value	*P* value
**Sex (n)**								
Male	5			4				
Female	4			5				
**Race or ethnicity (n)**								
White	5			7				
Asian	4			2				
**Age (years)**	49 ± 4			51 ± 4				
**Diabetes (Y/N)**	3/6			2/7				
**Dyslipidaemia (Y/N)**	5/4			5/4				
**Hypertension (Y/N)**	2/7			2/7				
**Liver biopsy histology (NAFLD/NASH)**	6/3			7/2				
**IHCL (%)**	20.9 ± 2.9	26.8 ± 3.9	0.012	22.6 ± 6.9	23.5 ± 6.8	0.635	0.020	0.082
**Body weight (kg)**	83.3 ± 4.4	83.2 ± 4.0	0.914	93.6 ± 7.6	93.9 ± 7.4	0.556	0.438	0.578
**BMI (kg/m** ^**2**^ **)**	29.5 ± 1.4	29.5 ± 1.4	0.966	31.5 ± 1.9	31.6 ± 1.9	0.377	0.696	0.620
**Fat mass (kg)**	26.8 ± 3.4	27.1 ± 3.3	0.524	35.3 ± 5.2	34.9 ± 5.3	0.485	0.931	0.302
**Fat free mass (kg)**	56.5 ± 3.7	56.1 ± 3.3	0.631	58.3 ± 5.0	59.1 ± 5.2	0.055+	0.748	0.341

Abbreviations: BMI, body mass index; IHCL, intrahepatocellular lipid; NAFLD, non‐alcoholic fatty liver disease; NASH, non‐alcoholic steatohepatitis.

+ = non‐parametric statistical analysis.

Detailed volunteer characteristics are presented in Table S1, File S1.

Data are expressed as mean ± SEM.

Whilst breath hydrogen, a marker of colonic fermentation, was elevated in both groups postsupplementation (Table S3, File S1), the impact on SCFAs measured in peripheral blood was limited, as we observed that IPE supplementation only reduced levels of butyrate in fasting samples compared to the inulin‐control group (Table S3, File S1). The blood samples were collected >12 hours after volunteers were requested to ingest their final supplement, which may explain why we were unable to detect large differences in circulating SCFAs postsupplementation. Nevertheless, previous research using stable isotope methodology has showed that inulin is predominantly fermented in the human colon into acetate (82%), with considerably less propionate and butyrate produced (6% and 12%, respectively).^10^ Dietary supplementation with inulin‐type fructans (ITF) has generally been associated with positive effects on metabolic health. The evidence for this beneficial effect is primarily derived from rodent studies, where dietary supplementation with ITF has consistently been shown to prevent the accumulation of liver fat and metabolic dysregulation induced by a high fat diet.^3^, ^4^, ^5^ However, to the best of our knowledge, ITF have not been shown to reduce liver fat when added to the diets of rodents with pre‐existing steatosis. Studies investigating the effect of ITF on metabolic health in humans are equivocal, with a recent meta‐analysis reporting no association between ITF supplementation and fasting glucose and insulin levels.^11^ Fewer studies have quantified the impact of ITF supplementation on liver fat content in humans. Our previous work showed that 30 g/d ITF supplemented into the habitual diet of overweight adults with normal glycaemic control had no effect on IHCL.^12^ In contrast, a superior reduction in IHCL in adults with prediabetes was found when a weight‐loss diet was combined with 30 g/d ITF supplementation.^13^ The serendipitous observation in the current study is that supplementing 20 g/d inulin into a habitual weight‐maintaining diet raises IHCL and further exacerbates glucose homeostasis in adults with NAFLD. Taken together, our data suggest that ITF supplementation does not have a homogenous impact on hepatic lipid content in humans, and its effects may depend on the pre‐existing metabolic health of the individual and the energy‐balance promoted by the background diet.

Previous research would suggest that the acetate derived from inulin fermentation would have contrasting metabolic fates depending on hepatic lipid metabolism in different physiological conditions. For example, it has previously been reported that greater amounts of exogenous acetate are used for hepatic de novo lipogenesis (DNL) in obese compared to lean individuals. This metabolic response was associated with higher insulin levels in the obese group, which is the chief regulator of hepatic DNL.^14^ Previous work has also showed that chronic intragastric acetate infusion in rats promotes postprandial hyperinsulinaemia and increases liver triglyceride content.^15^ The conversion of SCFAs into metabolic intermediates is initially determined by the acyl‐CoA synthetase short‐chain family members (ACSS).^16^ Human hepatocytes express the cytosolic isoform *ACSS2*, which has high specificity for acetate and increases the availability of acetyl‐CoA for lipid synthesis.^16^ Evidence highlights that, together with higher insulin levels,^17^ humans with NAFLD have an elevated expression of hepatic genes that favour fat accumulation, with increased expression of acetyl‐CoA carboxylase (ACC) and fatty acid synthase (FASN), which are key enzymes in hepatic DNL.^18^ Consequently, NAFLD patients are reported to have rates of DNL up to 3‐fold higher compared with BMI‐matched controls.^17^ The current data suggest that in body weight‐stable individuals with NAFLD, an increased supply of acetate to the liver from the colonic fermentation of inulin provides surplus acetyl‐CoA for DNL and hepatic lipid accretion. Interestingly, diet‐induced body weight loss in mice has been shown to markedly reduce insulin levels and rates of hepatic DNL,^19^ whilst the expression of hepatic DNL‐related genes is also reduced by states of chronic negative energy balance.^20^ This may explain the disparate effect of inulin supplementation on liver fat content in the current study compared with our previous investigation when inulin intervention was added to a hypocaloric diet that achieved a ~5% reduction in body weight.^13^

IPE supplementation did not significantly raise IHCL content, as observed within the inulin‐control group. The contrasting outcome could be because of differences in the amounts of acetate derived from inulin fermentation throughout the supplementation period, as the inulin‐control group were provided with a greater amount of inulin compared to IPE (20 g/d vs. 14.6 g/d). In vitro faecal fermentation profiles have previously showed, however, that comparable quantities of acetate are produced from equivalent doses of inulin and IPE.^9^ Interestingly, IPE does substantially alter the proportion of SCFAs produced, as the molar ratio of acetate, propionate and butyrate changes from 74:16:10 with inulin to 25:69:6 with an equivalent amount of IPE.^9^ It could be suggested that the elevated ratio of colonic propionate : acetate promoted by IPE supplementation may have prevented the accumulation of liver fat observed in the inulin‐control group by impairing hepatic acetate metabolism. This proposed mechanism is concordant with the results from Wolever and colleagues, who showed using stable isotope technology that propionate inhibits incorporation of colonic [1,2‐^13^C] acetate into plasma lipids in humans.^7^ Studies using rat hepatocytes have also highlighted that propionate inhibits lipid synthesis when acetate is a major source of acetyl‐CoA.^8^ Recent evidence has showed a third ACSS isoform, *ACSS3*, for which propionate is the preferred substrate over acetate, and which is highly expressed in the mitochondrial matrix of hepatocytes.^16^ *ACSS3* converts propionate to propionyl‐CoA allowing it to enter mitochondrial respiration through succinate and the TCA cycle.^16^ Elevating hepatic propionate metabolism would therefore increase competition with acetate for conversion into their CoA adducts at tissue level, which may reduce cytosolic acetyl‐CoA availability for DNL. This potential mechanism is supported by a recent observation that exposing HepG2 cells to elevated ratios of propionate : acetate increases the formation of heptadecanoic acid derived from propionyl‐CoA, which inhibits the synthesis of palmitate from acetyl‐CoA.^21^

The present study has a number of potential limitations, chiefly, the considerable variability in metabolic health of the recruited volunteers. Nevertheless, the individual change in IHCL postsupplementation was not associated with any baseline metabolic variable (Table S6, File S1) and significant differences between groups at baseline were only found in two outcome measures (cholesterol and LDL‐cholesterol). In addition, the inclusion criteria permitted a histological diagnosis of NAFLD within the previous 5 years, thus a volunteer's histological characterization could have changed in the timeframe between initial diagnosis and recruitment into the study. However, all volunteers exhibited a raised IHCL (>5%) when assessed at baseline (Table S1, File S1).

In conclusion, inulin consumed at 20 g/d increased IHCL in body weight‐stable adults with NAFLD, an effect not observed with IPE supplementation. We speculate that in the context of NAFLD and a hepatic metabolic profile that stimulates DNL, the acetate derived from colonic fermentation of inulin could provide an additional lipogenic precursor to the liver. The increased colonic delivery of propionate from IPE appears to attenuate this acetate‐mediated increase in IHCL, possibly by interfering with the availability of acetate‐derived acetyl‐CoA for DNL. Further work is warranted to explore how altering colonic SCFA production profiles modulates the metabolic pathways that govern hepatic lipid storage in humans. In particular, future research should determine how the hepatic metabolic processing of acetate and propionate changes in different states of energy balance, and how to make distinctions between NAFLD patients and healthy controls.

## Supporting information


**File S1.** A detailed methodology.
**Figure S1.** Recruitment and retention in the study. Inulin‐propionate ester (IPE).
**Figure S2.** The effects of 42 days of inulin control and inulin propionate ester (IPE) supplementation on postprandial A, glucose and B, insulin responses. (Data are expressed as mean ± SEM (n = 8 each group).
**Table S1.** Volunteer characteristics. Histological assessment of the liver from biopsy. Intra hepatocellular lipid (IHCL) content, alanine transaminase, HbA1c, metabolic comorbidities and medications at baseline.
**Table S2.** Changes in fasting and postprandial metabolic responses following 42 days of inulin‐control or inulin propionate ester (IPE) supplementation. Data are expressed as mean ± SEM or 95% CI.
**Table S3.** Changes in fasting and postprandial SCFA following 42 days of inulin‐control or inulin propionate ester (IPE) supplementation. Data are expressed as mean ± SEM or 95% CI.
**Table S4.** Changes in inflammatory markers following 42 days of inulin‐control or inulin propionate ester (IPE) supplementation. Data are expressed as mean ± SEM or 95% CI.
**Table S5.** Changes in self‐reported food intake, physical activity and gastrointestinal side‐effects following 42 days of inulin‐control or propionate ester (IPE) supplementation. Data are expressed as mean ± SEM or 95% CI.
**Table S6.** Correlations between baseline variables and the delta change (Δ) in IHCL following 42 days of inulin‐control or propionate ester (IPE) supplementation.
