# Supplementary material for: The effects of dietary supplementation with inulin and inulin‐propionate ester on hepatic steatosis in adults with non‐alcoholic fatty liver disease
Source: Diabetes Obes Metab. 2018 Sep 16;21(2):372–6. doi: 10.1111/dom.13500 (PMC6667894; doi:10.1111/dom.13500)
Supplement: Supplementary file 1 — File S1. A detailed methodology. Figure S1. Recruitment and retention in the study. Inulin‐propionate ester (IPE). Figure S2. The effects of 42 days of inulin control and inulin propionate ester (IPE) supplementation on postprandial A, glucose and B, insulin responses. (Data are expressed as mean ± SEM (n = 8 each group). Table S1. Volunteer characteristics. Histological assessment of the liver from biopsy. Intra hepatocellular lipid (IHCL) content, alanine transaminase, HbA1c, metabolic comorbidities and medications at baseline. Table S2. Changes in fasting and postprandial metabolic responses following 42 days of inulin‐control or inulin propionate ester (IPE) supplementation. Data are expressed as mean ± SEM or 95% CI. Table S3. Changes in fasting and postprandial SCFA following 42 days of inulin‐control or inulin propionate ester (IPE) supplementation. Data are expressed as mean ± SEM or 95% CI. Table S4. Changes in inflammatory markers following 42 days of inulin‐control or inulin propionate ester (IPE) supplementation. Data are expressed as mean ± SEM or 95% CI. Table S5. Changes in self‐reported food intake, physical activity and gastrointestinal side‐effects following 42 days of inulin‐control or propionate ester (IPE) supplementation. Data are expressed as mean ± SEM or 95% CI. Table S6. Correlations between baseline variables and the delta change (Δ) in IHCL following 42 days of inulin‐control or propionate ester (IPE) supplementation. [file DOM-21-372-s001.pdf]

SUPPLEMENTARY MATERIAL

METHODS

Potential participants were excluded if they met any of the following criteria: diagnosis of cirrhosis, other clinically significant illness, started new medication or changed the dose of existing medication in the preceding 3 months likely to interfere with energy metabolism, a weight loss of 3 kg or greater in the preceding two months, smoking, substance abuse, psychiatric illness, and any abnormalities detected on physical examination, electrocardiography, or screening blood tests (measurement of complete blood count, electrolytes and thyroid function). Women were ineligible if they were pregnant or breast-feeding. A urinary pregnancy test was conducted at screening and before the collection of experimental data at the two study visits.

A previous investigation found that 20 g/day IPE improved measures of metabolic health (fasting insulin and HOMA-IR) in overweight and obese adults at the end of a 42 day intervention (unpublished data). Liver fat content was not assessed in this investigation, but it was postulated the improvements in glucose homeostasis would be related to reductions in intrahepatocellular lipid (IHCL), as observed in previous work[1]. The same dose (20 g/day) and supplementation period (42 days) was therefore used in the current study.

Two strata were defined according to gender and randomisation sequences and allocation was conducted via a remote internet-based service (www.sealedenvelope.com). The supplement sachets were prepared by DJM and labelled ‘A’ and ‘B’. DJM held the key to the allocations for the duration of the trial. Throughout the trial, none of the participants or investigators involved in the trial had complete information on the randomisation allocations.

All subjects were instructed to maintain their usual dietary and activity habits during the study period and regular communication between subjects and study investigators encouraged good compliance. Subjects returned all their used and unused sachets to estimate compliance.

The day prior to the study visits, participants were requested to refrain from strenuous exercise and alcohol prior to fasting overnight for >12 hours. Participants were asked to eat the same ready-made or shop-prepared meal the evening before each study visit. Participants were free to choose their meal and compliance was assessed by requesting that volunteers bring the outer packaging of the meal and the receipt of purchase to the study visit. Participants were requested to ingest their final supplement sachet with their evening meal.

Liver fat, body weight and composition

Liver fat content was assessed using MRS, as previously described[2]. Body weight, fat mass (FM) and fat free mass (FFM) with bioelectrical impedance (Tanita BC-418MA, Japan). Subjects were

asked to change into lightweight hospital scrubs and to void their bladder before measurements were taken.

Mixed meal test (MMT)

A cannula was inserted into an antecubital vein and two fasting blood samples were collected >5 min apart to assess plasma concentrations of glucose and serum concentrations of insulin and short chain fatty acids (SCFA). At 0 min, subjects were served a standard liquid meal (Ensure Plus, Abbott, UK: 660 kcal; 88.9 g carbohydrate, 21.6 g fat, 27.5 g protein) that was ingested within 10 min. Postprandial blood samples were taken at 10, 20, 30, 45, 60, 90, 120 and 180 min and collected into sodium fluoride-coated tubes and serum-separating tubes. Plasma glucose was measured using an Abbott Architect ci8200 analyser (Abbott Diagnostics, USA). Insulin-like immunoreactivity was measured using an ultra-sensitive human insulin radioimmunoassay (Millipore, USA). SCFA were measured in fasting and the 60 min samples with the use of an Agilent 7000C Triple Quadrupole GC/MS System according to a previously published method [3]. Fasting breath hydrogen concentrations, a marker of colonic fermentation [4], were measured using a handheld breath hydrogen analyser (EC60 Gastrolyser Breath Hydrogen Monitor, Bedfont Scientific, Upchurch, Kent, UK).

Cardiovascular disease, diabetes risk factors and inflammatory markers.

A fasting blood sample was collected and analysed for levels of triglycerides, total cholesterol, low-density lipoprotein (LDL) cholesterol, high-density lipoprotein (HDL) cholesterol, glycosylated HbA1c, alanine transaminase and C-reactive protein. All analytes were measured by the Department of Chemical Pathology, Imperial College Healthcare National Health Service Trust. IL-6, IL-8, IL-10, IL-12 and IL-17A were measured in fasting serum using the Cytometric Bead Array (BD Biosciences, UK), according to the manufacturer's protocol. IL-17A results are not shown, as only two volunteers had detectable values for this analyte.

Self-reported food intake, physical activity and gastrointestinal adverse events

Energy and macronutrient intake was recorded with food diaries during the final 3-days of each supplementation period (Dietplan 6.0; Forestfield Software Ltd, UK). Physical activity was assessed during the final 7 days of each supplementation period using the short self-administered format of the International Physical Activity Questionnaire (IPAQ)[5]. Ratings of gastrointestinal side-effects were made using 100 mm visual analogue scales (VAS). Subjects were asked to rate the occurrence of each side effect with extreme statements anchored at each end of the rating scale (0 mm Never, 100 mm All the time) [1].

Calculations and statistical analysis

Data from our previous study was used to estimate the required sample size [1]. A power calculation confirmed that 16 participants (8 per group) would be sufficient to detect a mean 8% difference between groups in the baseline change in IHCL, with a common standard deviation (SD) of 5% (α=0.05, power=0.80). 20 volunteers were recruited to allow an estimated attrition rate of 20%. Time course data from the MMT were analysed by calculating areas under the curve (AUC) using the trapezoid rule and dividing by 180 to generate a mean postprandial value. Insulin resistance was assessed by homeostatic model assessment (HOMA-IR)[6].

Mixed analysis of variance was conducted to assess if the change in outcome measures was different over time (pre-supplementation, post-supplementation) between the two supplementation groups (inulin-control, IPE). The interaction (Group×Time) and main effect for time are presented. Levene's Test and Box's Test were performed to confirm homogeneity of variances. Within-group differences were compared using paired t tests. Data were checked for normality using the Shapiro-Wilk Test. Non-parametric data were log transformed prior to carrying out parametric statistical tests. Non-parametric within-group analysis (Wilcoxon signed rank test) was performed on data when log transformed values were not normally distributed. Between-group differences at baseline were assessed using unpaired t tests. Data were checked for normality using the Shapiro-Wilk Test. Non-parametric data were log transformed prior to carrying out parametric statistical tests. Non-parametric between-group analysis (independent samples Mann-Whitney U Tests) was performed on data when log transformed values were not normally distributed. Correlation analysis was performed to assess if the delta change (Δ) in liver fat content was related to baseline variables. Pearson correlation coefficients or Spearman's rank correlation coefficients were calculated All statistical analyses were carried out with SPSS version 23.0 for Windows (SPSS Inc, USA). Data are presented as means ± SEM or 95% CI. n = 9 per supplementation group, unless otherwise state. Due to difficulties cannulating two volunteers at post-supplementation visits, postprandial values from the mixed-meal test MMT were analysed from 16 volunteers (8 per supplementation group). $P<0.05$ was considered significant.

FIGURES

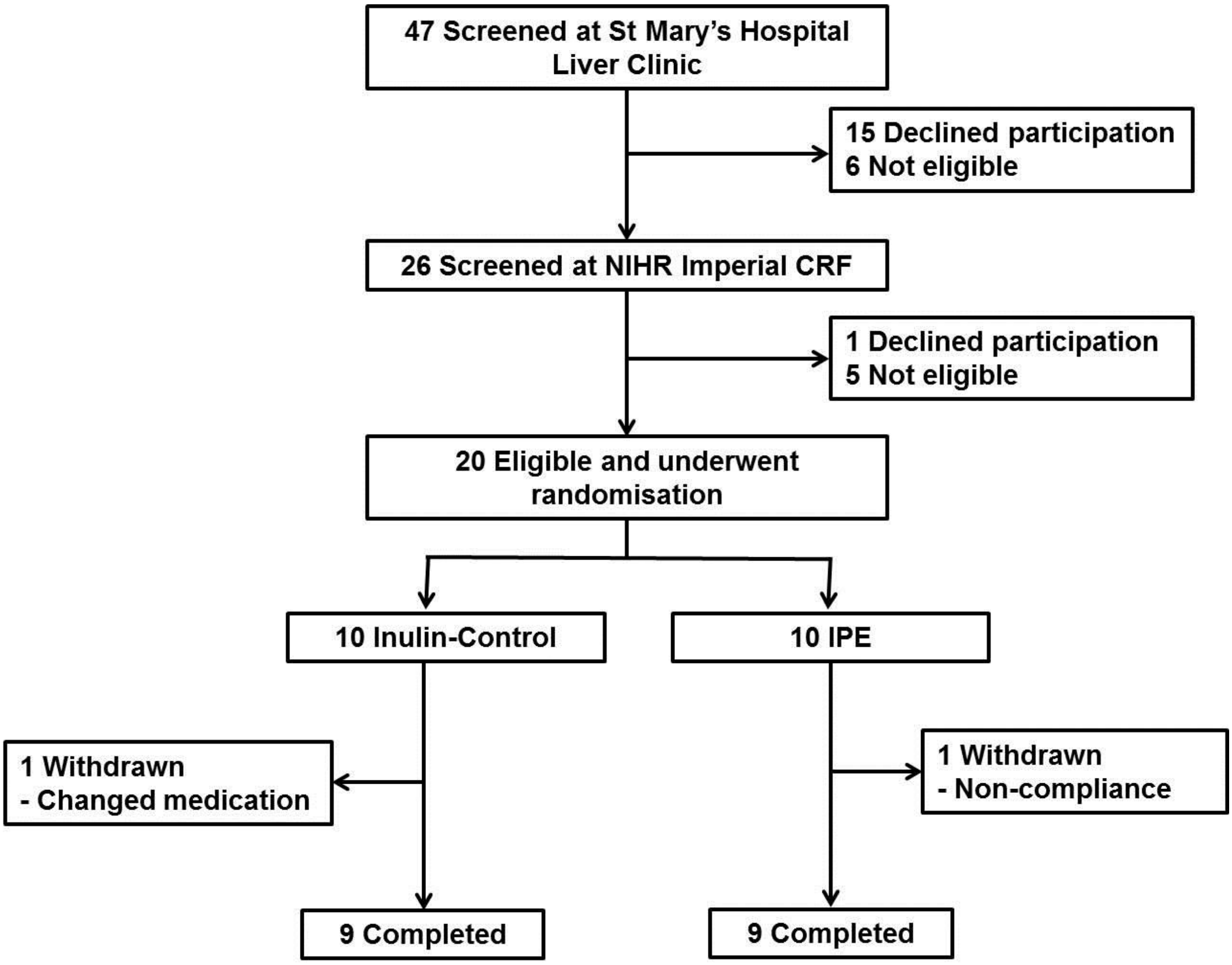

Figure S1. Recruitment and retention in the study. Inulin-propionate ester (IPE).

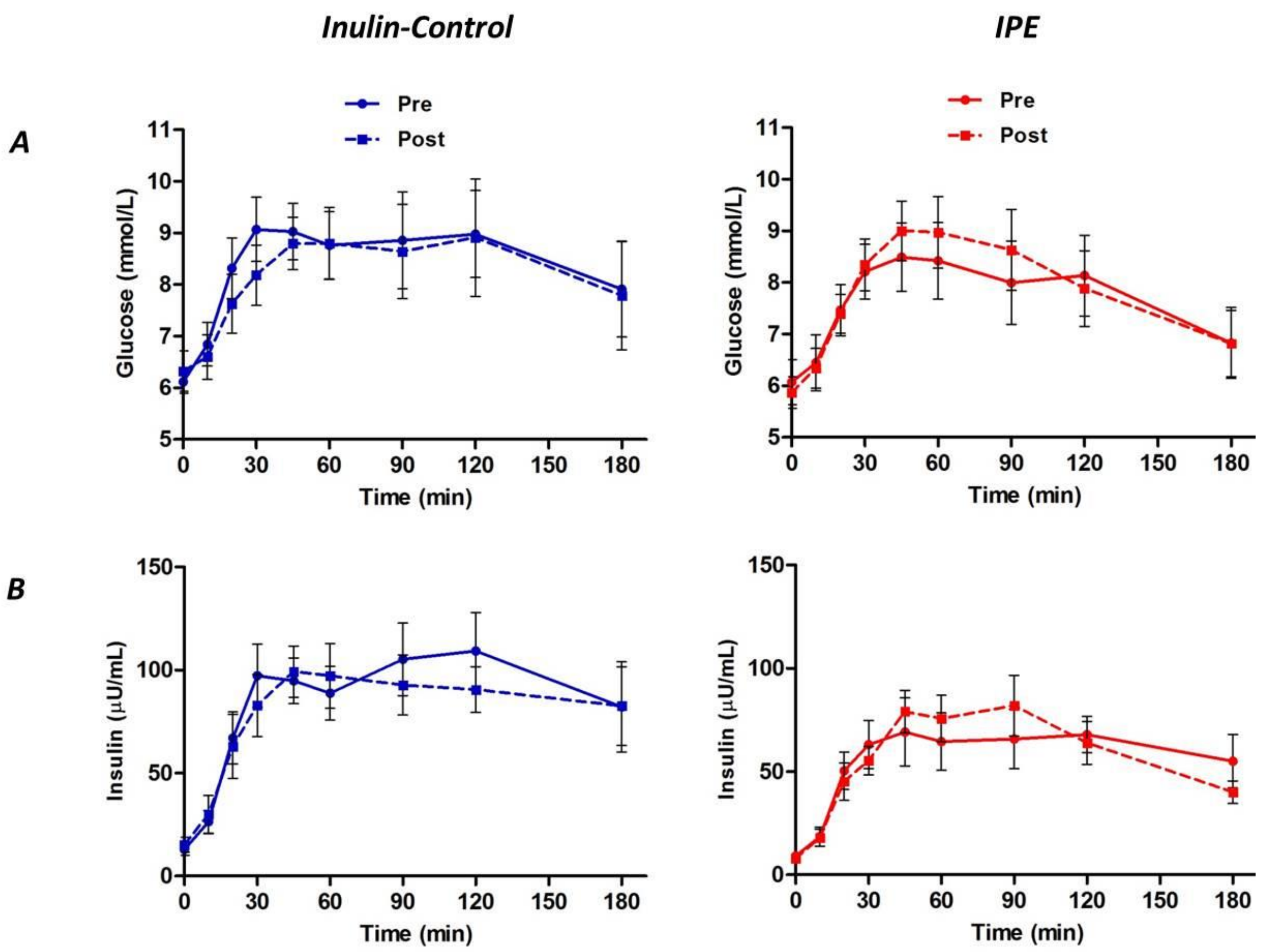

Figure S2. The effects of 42 days of inulin control and inulin propionate ester (IPE) supplementation on postprandial *A.* glucose and *B.* insulin responses (Data are expressed as mean ± SEM (n = 8 each group).

TABLES

Table S1. Volunteer characteristics. Histological assessment of the liver from biopsy. Intra hepatocellular lipid (IHCL) content, alanine transaminase, HbA1c, metabolic comorbidities and medications at baseline.

Volunteer	Group	Steatosis Grade^	Diagnostic Classification	Fibrosis	Years Between Biopsy and Recruitment	IHCL (%)	Alanine Transaminase (IU/L)	HbA1c (mmol/mol)	Type 2 Diabetes - Medication	Dyslipidaemia - Medication	Hypertension - Medication
**1**	IC	1	NAFLD	None	4	19	61	40	Y- Metformin	Y- Statins	Y- Bendroflumethiazide
**2**	IC	2	NASH	Mild	4	24	47	42	N	N	N
**3**	IC	1	NASH	None	2	34	123	42	N	N	N
**4**	IC	2	NAFLD	Not Specified	5	10	97	40	N	Y- Statins	N
**5**	IC	1	NAFLD	None	4	12	36	35	N	N	Y- Amlodipine
**6**	IC	2	NASH	Mild	3	29	47	48	Y- Metformin	Y- Statins	N
**7**	IC	1	NAFLD	None	4	15	30	37	N	N	N
**8**	IC	1	NAFLD	None	2	15	59	41	N	Y- Statins	N
**9**	IC	1	NAFLD	None	2	31	33	48	Y- Metformin	Y- Statins	N
**10**	IPE	1	NAFLD	None	3	24	30	32	N	N	N
**11**	IPE	1	NAFLD	Not Specified	4	25	57	37	N	Y- Statins	N
**12**	IPE	1	NASH	Mild	3	23	58	48	Y- Metformin	Y- Statins	Y- Amlodipine
**13**	IPE	1	NAFLD	None	2	5	29	35	N	N	N
**14**	IPE	2	NASH	None	4	53	122	41	N	N	N
**15**	IPE	1	NAFLD	None	3	5	19	42	N	Y- Statins	Y- Ramipril
**16**	IPE	1	NAFLD	Not Specified	5	6	11	36	N	N	N
**17**	IPE	1	NAFLD	None	3	15	25	46	Y- Metformin	Y- Statins	N
**18**	IPE	2	NASH	None	3	46	37	48	N	Y- Statins	N

Inulin-control (IC), inulin-propionate ester (IPE), non-alcoholic fatty liver disease (NAFLD), non-alcoholic steatohepatitis (NASH), Glycosylated haemoglobin (HbA1c).
^Steatosis graded: 0=<5%, 1=5-33%, 2=33-66%, 3=>66%[7].

Table S2. Changes in fasting and postprandial metabolic responses following 42 days of inulin-control or inulin propionate ester (IPE) supplementation. Data are expressed as mean ± SEM or 95% CI.

Variable	Inulin-Control (N = 9)			IPE (N = 9)			Mixed ANOVA	
	Pre	Post	P Value	Pre	Post	P Value	Time P Value	Group×Time P Value
Fasting Glucose (mmol/L)	6.1 ± 0.2	6.3 ± 0.4	0.343	6.1 ± 0.4	5.9 ± 0.3	0.211	0.943	0.190
Postprandial Glucose^ (mmol/L)	8.5 ± 0.7	8.3 ± 0.8	0.610	7.8 ± 0.7	7.9 ± 0.6	0.431	0.897	0.416
Fasting Insulin (μU/mL)	12.9 ± 2.8	15.3 ± 3.5	0.102	9.1 ± 2.0	7.9 ± 1.7	0.496	0.616	0.115
Postprandial Insulin^ (μU/mL)	89.9 ± 12.5	83.67 ± 11.2	0.409	59.4 ± 9.7	59.2 ± 7.7	0.967	0.558	0.494
HOMA-IR	3.6 ± 0.9	4.5 ± 1.2	0.060	2.6 ± 0.7	2.2 ± 0.6	0.389	0.418	0.046
HbA1c (mmol/mol)	41.9 ± 3.7	43.5 ± 2.3	0.056	41.5 ± 2.6	41.3 ± 2.5	0.821	0.203	0.113
Triglycerides (mmol/L)	1.1 ± 0.1	1.9 ± 0.9	0.148+	1.4 ± 0.3	1.5 ± 0.2	0.842	0.227	0.564
Cholesterol† (mmol/L)	4.0 ± 0.3	4.0 ± 0.4	0.957	5.4 ± 0.3	5.3 ± 0.4	0.510	0.650	0.597
LDL Cholesterol† (mmol/L)	2.4 ± 0.2	2.1 ± 0.3	0.303	3.5 ± 0.3	3.4 ± 0.4	0.181	0.143	0.606
HDL Cholesterol (mmol/L)	1.1 ± 0.1	1.1 ± 0.1	0.778	1.2 ± 0.1	1.2 ± 0.1	0.813	0.989	0.715
Alanine Transaminase (IU/L)	59.2± 10.5	67.6 ± 9.4	0.290	43.1 ± 11.2	44.0 ± 11.1	0.718	0.235	0.382
Breath Hydrogen (ppm)	10.5± 2.9	21.1 ± 5.4	0.014	3.7 ± 0.8	11.4 ± 3.8	0.028	0.001	0.651

+ = non-parametric statistical analysis. ^ =statistical analysis performed on n=8 each group. † = Significant difference between groups at baseline. Homeostatic model assessment of insulin resistance (HOMA-IR), Glycosylated haemoglobin (HbA1c), Low density lipoprotein (LDL), High density lipoprotein (HDL).

Table S3. Changes in fasting and postprandial SCFA following 42 days of inulin-control or inulin propionate ester (IPE) supplementation. Data are expressed as mean ± SEM or 95% CI.

	Inulin-Control (N = 9)			IPE (N = 9)			Mixed ANOVA	
**Variable**	**Pre**	**Post**	**P Value**	**Pre**	**Post**	**P Value**	**Time P Value**	**Group×Time P Value**
**Fasting Acetate (μmol/L)**	16.5 ± 2.5	19.7 ± 3.7	0.562	25.4 ± 2.8	35.8 ± 5.5	0.157	0.128	0.409
**Postprandial Acetate^ (μmol/L)**	20.4 ± 2.0	24.0 ± 4.1	0.376	25.5 ± 4.3	25.7 ± 4.4	0.955	0.475	0.522
**Fasting Propionate (μmol/L)**	2.1 ± 0.1	2.4 ± 0.3	0.336	2.6 ± 0.3	2.5 ± 0.3	0.847	0.605	0.425
**Postprandial Propionate^ (μmol/L)**	2.5 ± 0.3	2.4 ± 0.4	0.640	2.5 ± 0.2	3.2 ± 0.4	0.117	0.262	0.155
**Fasting Butyrate (μmol/L)**	1.7 ± 0.2	1.9 ± 0.2	0.506	2.5 ± 0.3	2.0 ± 0.2	0.003	0.214	0.023
**Postprandial Butyrate^ (μmol/L)**	2.1± 0.2	2.2 ± 0.2	0.891	1.9 ± 0.2	2.2 ± 0.3	0.548	0.529	0.640

^ =statistical analysis performed on n=8 each group.

Table S4. Changes in inflammatory markers following 42 days of inulin-control or inulin propionate ester (IPE) supplementation. Data are expressed as mean ± SEM or 95% CI.

Variable	Inulin-Control (N = 9)			IPE (N = 9)			Mixed ANOVA	
							Time	Group×Time
	Pre	Post	P Value	Pre	Post	P Value	P Value	P Value
IL-6 (pg/mL)	5.4 ± 3.7	3.0 ± 1.2	0.438+	1.9 ± 0.5	1.7 ± 0.3	0.500+	0.293	0.541
IL-8 (pg/mL)	7.7 ± 1.6	11.0 ± 4.6	0.610	6.1 ± 1.1	5.8 ± 1.0	0.650	0.942	0.973
IL-10 (pg/mL)	2.0 ± 0.7	3.0 ± 1.2	0.393+	1.6 ± 0.3	1.4 ± 0.1	0.351+	0.592	0.269
IL-12 (pg/mL)	28.8 ± 6.8	39.5 ± 11.6	0.098+	18.4 ± 3.0	19.6 ± 3.4	0.513	0.168	0.342
C Reactive Protein (mg/L)	2.3 ± 0.7	3.1 ± 1.5	0.672+	5.8 ± 2.8	5.7 ± 2.9	0.867+	0.839	0.920

+ = non-parametric statistical analysis

Table S5. Changes in self-reported food intake, physical activity and gastrointestinal side-effects following 42 days of inulin-control or propionate ester (IPE) supplementation. Data are expressed as mean ± SEM or 95% CI.

	Inulin-Control (N = 9)			IPE (N = 9)			Mixed ANOVA	
							Time	Group×Time
**Variable**	Pre	Post	P Value	Pre	Post	P Value	P Value	P Value
**Energy Intake (kcal/day)**	2418 ± 381	2044 ± 235	0.242	2233 ± 254	2268 ± 141	0.878	0.367	0.281
**Carbohydrate (g/day)**	275 ± 40	237 ± 25	0.216	248 ± 32	255 ± 24	0.796	0.521	0.185
**Fat (g/day)**	112 ± 23	89 ± 13	0.284	93 ± 14	94 ± 8	0.945	0.378	0.338
**Protein (g/day)**	95 ± 11	85 ± 9	0.260	101 ± 8	98 ± 5	0.709	0.281	0.589
**Fibre (NSP) (g/day)**	17 ± 3	14 ± 2	0.334	14 ± 1	13 ± 1	0.321	0.178	0.689
**Total Physical Activity (MET-h/week)**	13 ± 3	19 ± 6	0.412	19 ± 6	21 ± 5	0.673	0.349	0.636
**Stomach Discomfort (mm)**	25 ± 10	28 ± 9	0.818	9 ± 7	6 ± 2	0.638	0.864	0.909
**Nausea (mm)**	11 ± 6	24 ± 10	0.358	11 ± 7	3 ± 1	0.234	0.849	0.154
**Bloating (mm)**	22 ± 11	33 ± 12	0.519	9 ± 4	16 ± 5	0.352	0.282	0.949
**Flatulence (mm)**	20 ± 10	59 ± 8	0.006	17 ± 8	25 ± 10	0.591	0.020	0.094
**Heartburn (mm)**	13 ± 6	5 ± 3	0.292	12 ± 5	3 ± 1	0.145	0.091	0.742
**Belching (mm)**	18 ± 10	17 ± 9	0.968	7 ± 4	4 ± 2	0.643	0.870	0.956

Energy intake was recorded with 3-day food diaries. Physical activity was measured using the short self-administered format of the International Physical Activity Questionnaire (IPAQ) [5]. Ratings of gastrointestinal side-effects were made using 100 mm visual analogue scales (VAS). Subjects were asked to rate the occurrence of each side effect with extreme statements anchored at each end of the rating scale (0 mm Never, 100 mm All the time).

Table S6. Correlations between baseline variables and the delta change (Δ) in IHCL following 42 days of inulin-control or propionate ester (IPE) supplementation.

Baseline Variable	ΔIHCL (%) Inulin-Control (N=9)		ΔIHCL (%) IPE (N=9)		ΔIHCL (%) All (N=18)	
	Pearson Correlation	P Value	Pearson Correlation	P Value	Pearson Correlation	P Value
IHCL (%)	0.295	0.440	-0.259	0.501	0.138	0.624
Fasting Glucose (mmol/L)	0.186	0.186	-0.247	0.556	-0.071	0.802
Postprandial Glucose (mmol/L)^	-0.076	0.858	-0.178	0.674	-0.095	0.736
Fasting Insulin (µU/mL)	-0.291	0.484	0.192	0.650	0.062	0.825
Postprandial Insulin (µU/mL)^	-0.014	0.974	0.488	0.220	0.075	0.790
HOMA-IR	-0.206	0.625	0.048	0.910	0.050	0.860
HbA1c (mmol/mol)	0.089	0.819	-0.592	0.093	-0.170	0.545
Triglycerides (mmol/L)	-0.447	0.227	-0.053	0.893	-0.211+	0.451
Cholesterol (mmol/L)	-0.207	0.593	-0.453	0.221	-0.266	0.338
LDL Cholesterol (mmol/L)	-0.106	0.786	-0.323	0.397	-0.156	0.579
HDL Cholesterol (mmol/L)	-0.216	0.576	-0.276	0.473	-0.185	0.510
Alanine Transaminase (IU/L)	0.385	0.306	0.134	0.731	0.206	0.462
Breath Hydrogen (ppm)	0.490	0.218	-0.481	0.227	0.074+	0.794
Fasting Acetate (µmol/L)	0.053	0.901	0.128	0.763	0.082+	0.771
Postprandial Acetate (µmol/L)^	0.614	0.105	0.297	0.475	0.303	0.273
Fasting Propionate (µmol/L)	-0.086	0.839	-0.412	0.310	-0.258	0.353
Postprandial Propionate (µmol/L)^	0.601	0.115	0.207	0.623	0.416	0.109
Fasting Butyrate (µmol/L)	-0.174	0.728	-0.266	0.524	-0.411	0.128
Postprandial Butyrate (µmol/L)^	-0.338	0.413	0.642	0.086	-0.041	0.885
IL-6 (pg/mL)	-0.050+	0.898	-0.365	0.374	-0.279+	0.315
IL-8 (pg/mL)	0.007	0.986	-0.193	0.647	-0.161	0.566
IL-10 (pg/mL)	0.067+	0.864	-0.578+	0.133	-0.153+	0.586
IL-12 (pg/mL)	0.337	0.318	-0.051	0.904	-0.088+	0.756
C Reactive Protein (mg/L)	0.567	0.112	0.233	0.615	0.323	0.022
Energy Intake (kcal/day)	0.556	0.153	0.037	0.930	0.411	0.128
Carbohydrate (g/day)	0.512	0.195	0.452+	0.260	0.439+	0.101
Fat (g/day)	0.552	0.156	-0.034	0.937	0.411	0.128
Protein (g/day)	0.478	0.231	-0.090	0.832	0.232	0.406
Fibre (NSP) (g/day)	0.243	0.563	0.162	0.701	0.276	0.319
Total Physical Activity (MET-h/week)	-0.405	0.280	-0.076	0.846	-0.428	0.111

+ = non-parametric Spearman’s Rank correlation coefficient. ^ =statistical analysis performed on n=8/n=16.

REFERENCES

1. Chambers ES, Viardot A, Psichas A, et al. Effects of targeted delivery of propionate to the human colon on appetite regulation, body weight maintenance and adiposity in overweight adults. *Gut.* Nov 2015;64(11):1744-1754.

2. Thomas EL, Parkinson JR, Frost GS, et al. The missing risk: MRI and MRS phenotyping of abdominal adiposity and ectopic fat. *Obesity (Silver Spring).* Jan 2012;20(1):76-87.

3. Moreau NM, Goupry SM, Antignac JP, et al. Simultaneous measurement of plasma concentrations and 13C-enrichment of short-chain fatty acids, lactic acid and ketone bodies by gas chromatography coupled to mass spectrometry. *J Chromatogr B Analyt Technol Biomed Life Sci.* Feb 05 2003;784(2):395-403.

4. Levitt MD. Production and excretion of hydrogen gas in man. *N Engl J Med.* Jul 17 1969;281(3):122-127.

5. Craig CL, Marshall AL, Sjostrom M, et al. International physical activity questionnaire: 12-country reliability and validity. *Med Sci Sports Exerc.* Aug 2003;35(8):1381-1395.

6. Matthews DR, Hosker JP, Rudenski AS, Naylor BA, Treacher DF, Turner RC. Homeostasis model assessment: insulin resistance and beta-cell function from fasting plasma glucose and insulin concentrations in man. *Diabetologia.* Jul 1985;28(7):412-419.

7. Kleiner DE, Brunt EM, Van Natta M, et al. Design and validation of a histological scoring system for nonalcoholic fatty liver disease. *Hepatology.* Jun 2005;41(6):1313-1321.
